# A One-Bead-Per-Saccharide (1BPS) Model for Glycosaminoglycans

**DOI:** 10.1021/acs.jctc.3c00238

**Published:** 2023-07-17

**Authors:** Saber Shakibi, Patrick R. Onck, Erik Van der Giessen

**Affiliations:** Micromechanics of Materials, Zernike Institute for Advanced Materials, University of Groningen, 9747 AG Groningen, The Netherlands

## Abstract

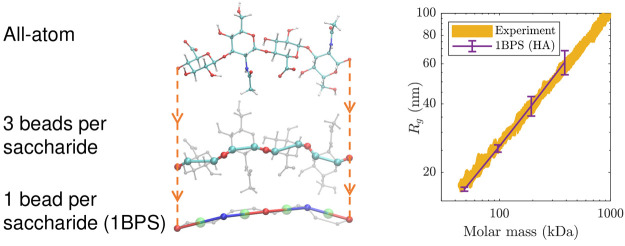

Glycosaminoglycans (GAGs) are polysaccharide compounds that play key roles in various biological processes. GAGs are important structural components of cartilage and the extracellular matrix of the brain. Due to the large size of these polysaccharides, coarse-grained approaches are indispensable for modeling these biopolymers. We develop a one-bead-per-saccharide model of chondroitin sulfates and hyaluronic acid based on an existing three-bead-per-saccharide coarse-grained model. Our coarse graining is carried out by using iterative Boltzmann inversion (IBI), including an additional coupling potential to incorporate the correlation between dihedral angles. The predictions of the model are verified against those of the existing three-bead-per-saccharin model and the experimental radius of gyration for hyaluronic acid.

## 1 Introduction

Nature employs a large variety of biopolymers including proteins, nucleic acids, and polysaccharides. Glycosaminoglycans (GAGs) are a class of unbranched polysaccharides that comprise hyaluronic acid (HA), chondroitin sulfates (CSs), dermatan sulfates, keratan sulfates, and heparan sulfates. GAGs play functional roles in various biological processes^1^ including angiogenesis,^2^ inflammation, cancer,^3,4^ cell penetration,^5^ neurogenesis, neuronal plasticity, and wound healing.^6^

GAGs also play important structural roles in the body: they connect to core proteins to form proteoglycans which form aggregates with HA to provide low-friction load-bearing properties of cartilage.^7^ Similar aggregates serve as the backbone of the extracellular matrix (ECM) of the brain.^8,9^ Perineuronal nets are the most simple compartments of the brain ECM in terms of the number of components: they comprise HA, CS proteoglycans, and tenascins.^8^ Expression levels of all of these components are changed in cancer^10,11^ leading to remodeling of the brain ECM. This remodeling is known to have an important role in tumor progression.^12^ Our long-term aim is to develop a computational multiscale model to study the relationship between this remodeling and the mechanical properties of the brain ECM; the present work is a key step toward that goal.

Due to the large size of proteoglycans^13^ and HA in the brain (with molecular weights of ∼1 MDa),^12^ atomistic models cannot be used for these biopolymer complexes. Therefore, coarse-grained (CG) models are needed that are coarse enough to allow for modeling these biopolymers. The one-bead-per-amino acid (1BPA) model developed for disordered proteins^14,15^ is a good candidate for modeling the core protein of proteoglycans as it is mostly composed of disordered regions. The 1BPA model has been used for modeling FG-Nups in the nuclear pore complex and for studying phase separation of toxic dipeptide repeats.^15−17^ Our aim is to develop a CG model to represent HA and CS chains that is compatible with the 1BPA model.

A number of CG models have been proposed for GAGs.^18−20^ Bathe et al.^18^ proposed a CG model of GAGs in which each monosaccharide is modeled by three beads immersed in an implicit solvent. Their predictions are close to experimental observations for chains with hundreds of monosaccharides. Samsonov et al.^19^ proposed a CG model in which each functional group is represented by a bead, leading to three to five beads for each monosaccharide. They developed their model for both implicit and explicit solvent. However, the model by Samsonov et al.^19^ tends to overestimate the radius of gyration of heparin chains having more than 36 monosaccharides (Table S8 of their work^19^). Kumar et al.^20^ developed a MARTINI CG model for HA. In their model, each monosaccharide is modeled by three beads and the solvent is treated explicitly. Due to the high computational cost of treating the solvent explicitly, they could not directly compare their results to experimental observations. Moreover, in view of the future application to proteoglycans, the spatial and temporal scales of a MARTINI model are incompatible with a 1BPA representation of proteins.

In all the above CG models for GAGs, a monosaccharide is modeled by three beads or more. This fine resolution is inconsistent with that of the 1BPA^14^ model in which a complete residue is represented by a single bead. With a view toward modeling proteoglycans, we here propose a one-bead-per-saccharide (1BPS) model. Our point of departure is the model by Bathe et al.^18^ because (1) it uses ingredients that are compatible with the 1BPA model, e.g., implicit solvent and a Debye screening electrostatic potential; (2) it accurately predicts the persistence length and radius of gyration of GAGs with lengths up to hundreds of monosaccharides (corresponding to a molecular weight of roughly hundreds of kDa) at different salt concentrations. Our 1BPS model will be developed for hyaluronic acid (HA), chondroitin-4-sulfate (C4S), and chondroitin-6-sulfate (C6S), which are the main GAGs involved in the proteoglycan aggregates in perineuronal nets. The procedure is presented in sufficient detail to guide the reader in developing force fields for other GAGs.

In section 2, we take a detailed look at the structure of GAGs and the definition of coarse-grained beads by Bathe et al.^18^ We then develop an MD version of the Monte Carlo model by Bathe et al.^18^ which is used as a fine-scale reference for developing the 1BPS model. Next, the 1BPS model is presented, followed by a discussion of the importance of dihedral coupling and the approximations made. In section 3, we describe the implemented iterative Boltzmann inversion (IBI) method, the procedure for incorporating a dihedral coupling potential, and the nonbonded interaction potentials. The effect of the coupling potential on the correlation between dihedrals is demonstrated in section 4. It is also shown that the predictions of the 1BPS model match those reported by Bathe et al.^18^ and other experimental observations. The article is concluded with a brief summary and outlook for other GAGs and polymers that can be coarse-grained using the methodology described here.

## 2 Approach

**Figure 1:**
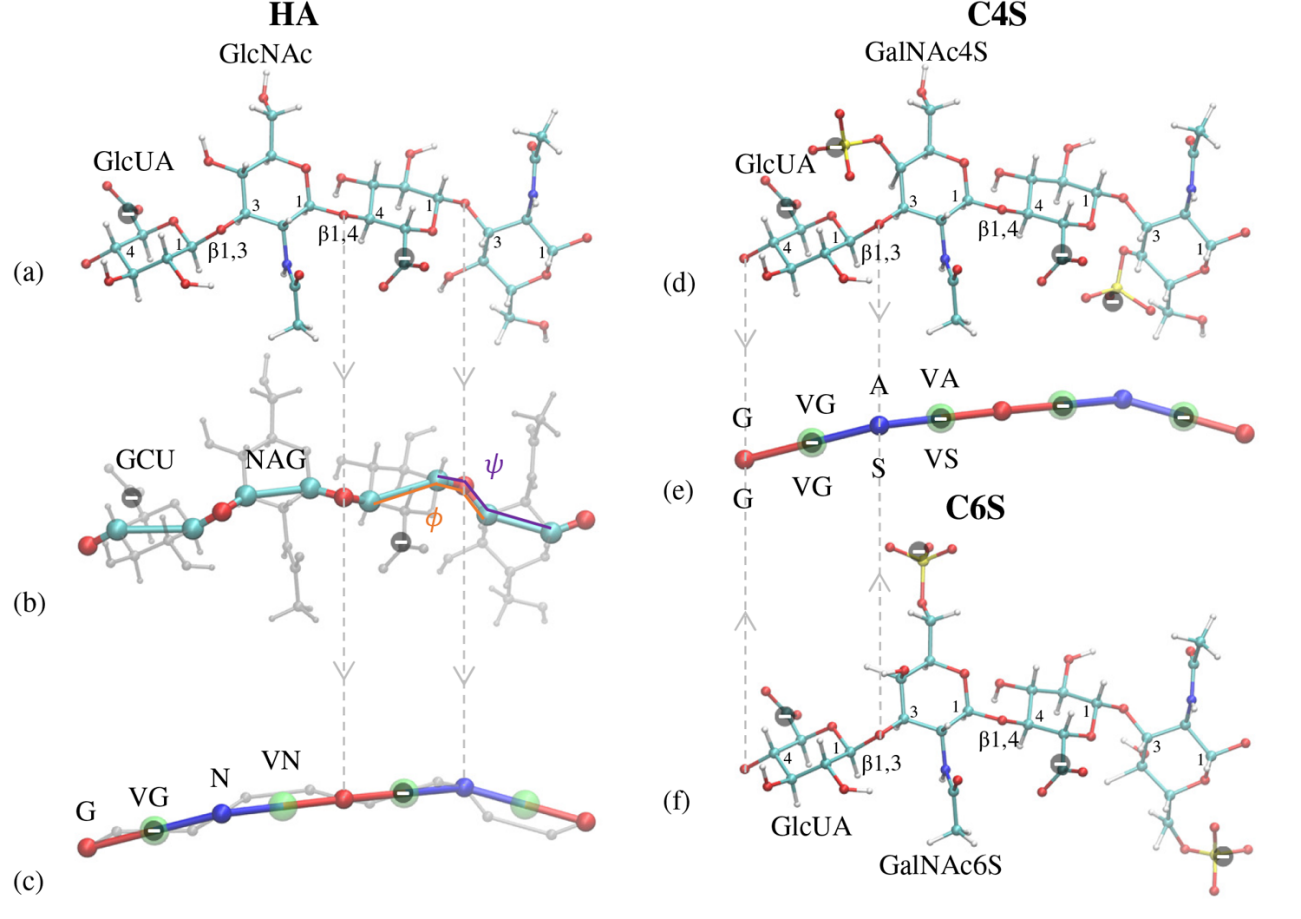
Schematic representation of the following: (a) Atomistic structure of HA with the approximate location of localized charges (shown by a negative sign inside a black circle) and types of glycosidic linkages. Carbon atoms are in cyan, oxygens in red, hydrogen in white and nitrogen in blue. (b) The model by Bathe et al.^18^ Beads located at carbon atoms are shown in cyan, and oxygen beads are shown in red. The glycosidic dihedral angles ψ and ϕ are shown in pink and orange, respectively. (c) The current (1BPS) model for HA. The blue and red beads define the backbone structure of the beads in the present model which are located on the oxygens in the glycosidic linkage. The transparent green spheres are virtual sites located in the middle of the two adjacent beads. These virtual sites are used to model nonbonded electrostatic and steric interactions. The letter G refers to the beads located on the β1,4 linkage preceding a GCU unit, while N refers to the beads located on the β1,3 linkage preceding a NAG unit. (d) Atomistic structure of C4S; sulfur atoms are shown in yellow. (e) 1BPS model of C4S and C6S. (f) Atomistic structure of C6S. The gray arrows highlight the transitions from fine-scale to coarser models.

Hyaluronic acid is a polysaccharide that consists of repeating disaccharide units of d-glucuronic acid (GlcUA) and *N*-acetyl-d-glucosamine (GlcNAc) that are linked by alternating β1,3 and β1,4 linkages (see Figure 1a). A β1,3 linkage connects a C1 atom in one monosaccharide to a C3 atom in the neighboring monosaccharide by a β type glycosidic linkage; similarly a β1,4 linkage connects a C1 to a C4. Chondroitin sulfates have similar disaccharide units but with *N*-acetyl-d-galactosamine being sulfated at the 4- or 6-carbon, also known as GalNAc4S and GalNAc6S, respectively. In all these GAGs, the average distance between adjacent glycosidic linkages is 5.25 Å. The GlcUA, GlcNAc, GalNAc4S and GalNAc6S monosaccharide units are sometimes designated by their three letter name as GCU, NAG, ASG, and NG6, respectively.

In the model by Bathe et al.,^18^ shown in Figure 1b, each monosaccharide is represented by two beads coinciding with the two carbon atoms adjacent to the glycosidic linkages (shown in cyan) and one bead at the position of the linking oxygen (shown in red). All the bonded interactions in this model are considered rigid except for the glycosidic dihedrals ψ and ϕ (shown in Figure 1b) in each linkage, for which Bathe et al.^18^ have reported glycosidic dihedral potentials. The backbone beads do not contribute to the nonbonded interactions; instead, these interactions are modeled via two virtual sites: one at the center of charge (shown by a minus sign in a black circle in Figure 1b) for electrostatic interactions and one at the center of geometry for steric interactions (not shown).

In this article, we propose a one-bead-per-saccharide (1BPS) model, shown in Figure 1c,e. In this model, the backbone beads (shown in red and blue) are located on the glycosidic oxygens and the interaction potentials between them are obtained by using iterative Boltzmann inversion on the basis of the results by Bathe et al.^18^ Backbone beads do not contribute to the nonbonded interactions; instead, electrostatic and steric interactions are modeled by virtual sites (transparent green spheres) at the center between two adjacent beads.

### 2.1 MD-Bathe Model

Bathe et al.^18^ have used Monte Carlo simulations to predict the conformation of isolated GAGs. In contrast, the 1BPA model^14^ is a Langevin molecular dynamics model. To make the models compatible, we developed a MD version of the model by Bathe et al.^18^ which we refer to as “MD-Bathe”. The latter required two modifications in the bonded interactions relative to the original model: (1) The glycosidic dihedral potential *V*(ψ, ϕ) in their model is a function of the two glycosidic dihedrals, yet most MD software packages (including GROMACS used here) require a single-variable potential. Therefore, each of these two-variable potentials needed to be translated into two single-variable potentials. (2) The rigid bonded interactions in the model by Bathe et al.^18^ needed to be replaced by “stiff” potentials with the same equilibrium value. Electrostatic and steric interactions are modeled by virtual sites at the same location and with the same potentials as adopted by Bathe et al.^18^

To translate the two-variable dihedral potentials *V*(ψ, ϕ) into single-variable potentials *V*(ψ) and *V*(ϕ), we first converted *V*(ψ, ϕ) into the joint probability distribution *P*(ψ, ϕ) = exp(−*V*(ψ, ϕ)/(*k*_B_*T*)), where *k*_B_ is Boltzmann’s constant and *T* is temperature (300 K). If it is assumed that the dihedral angles ψ and ϕ are independent, the probability distribution of the angle ψ is  and similar for *P*(ϕ). Using Boltzmann inversion, we then obtained the potential *V*(ψ) = −*k*_B_*T* ln *P*(ψ) and similarly the potential *V*(ϕ) for the dihedral angle ϕ. Following this proceedure, we obtained a maximum potential of ∼40*k*_B_*T* for *V*(ψ) and *V*(ϕ). However, relaxation simulations carried out for an HA chain with 128 monosaccharides using these potentials revealed that the maximum potential was too high to allow the MD-based model to sample all of the low energy states at *T* = 300 K within a time period of 500 ns. Therefore, we maximized the potentials *V*(ψ) and *V*(ϕ) to different values, as exemplified in Figure S1. As demonstrated in Figure S2, a maximum potential of 20*k*_B_*T* was found to result in HA chain conformations that match the reported values by Bathe et al.^18^ The dihedral potentials thus obtained for the MD-Bathe model of HA, C4S, and C6S are shown in Figure S3.

Except for the glycosidic linkage, all the distances, angles, and dihedrals are assumed to be rigid with a distance or angle reported in Table 2 of the Supporting Information of ref (18). The rigid bonds and angles were replaced with harmonic springs in the MD version. In order to select a proper value of the spring constants, we performed a sensitivity analysis of the normalized end-to-end distance (which will be introduced in section 4.1) for a HA chain with 128 monosaccharides. This study was carried out to (1) ensure that the chosen values for the spring constant are sufficiently high to reproduce the rigid-bond results by Bathe et al.^18^ and (2) validate that decoupling the potentials *V*(ψ, ϕ) into *V*(ψ) and *V*(ψ) described previously does not effect the predicted results. We confirmed that the chosen set of potentials (described in Supporting Information) reproduce the results of Bathe et al.^18^ In absence of nonbonded interactions, the mean squared end-to-end distance of HA, C4S, and C6S chains with 128 monosaccharides are 838, 1077, and 842 nm^2^, respectively, according to the MD-Bathe model. These values are in agreement with the Monte Carlo data reported by Bathe et al.^18^ However, the use of high spring constants restricts the time step of MD-Bathe simulations to only 1 fs. This is incompatible with the 20 fs time step used in the 1BPA model.^14^

### 2.2 1BPS Model

The assumption by Bathe et al.^18^ that all bonded interactions within a monosaccharide are rigid suggests that a monosaccharide may be regarded as a single bead. Here we propose such a 1BPS model in which a single bead, coinciding with the glycosidic oxygen, represents a monosaccharide (Figures 1c,e). For simplicity, we will call the bead located at the β1,4 linkage connected to a GCU monosaccharide a G bead; similarly, we will refer to the bead located at the β1,3 linkage connected to a NAG, ASG, and NG6 monosaccharide as N, A, and S beads, respectively. Virtual sites in the middle of two adjacent beads are used to incorporate the nonbonded (steric and electrostatic) interactions of each monosaccharide. These virtual sites are named VG, VN, VA, and VS, corresponding to the GCU, NAG, ASG, and NG6 monosaccharides, respectively. We use the letter X as a generic letter for N, A, or S beads, such that all GAGs in this paper can be considered to be polysaccharides with GX disaccharide repeats. The degrees of freedom (DOFs) involved in the bonded interactions are identified by the beads that define them; thus, for a general GAG, the DOFs consist of GX and XG bonds, GXG and XGX angles, and GXGX and XGXG dihedrals.

Because of the rigid-bond assumption by Bathe et al.,^18^ the distance between two adjacent glycosidic oxygens is fixed at 5.6 and 4.9 Å for GX and XG bonds, respectively. In the 1BPS model, the bonds GX and XG are governed by harmonic springs with a large spring constant of 8038 kJ mol^–1^ nm^–2^ just like the bond stretching stiffness in the 1BPA model.^14^

Coarse-graining implies a loss of information. Since the 1BPS model only includes beads at glycosidic oxygens, the exact location of virtual sites cannot be matched with the MD-Bathe model. However, we observed a nonsignificant 1% reduction in the persistence length of C4S chains in *C*_s_ = 150 mM when we changed the location of the virtual sites to the middle of the oxygens in the MD-Bathe model. Motivated by this observation, we decided to relocate the virtual sites to the midpoints between two neighboring oxygen atoms in the MD-Bathe model. Additionally, there was an implicit coupling between GXGX and XGXG dihedrals in the MD-Bathe model that was lost in the coarse-graining step. Since this coupling is essential for accurate prediction of the conformation of GAGs, a “coupling” potential was incorporated in the 1BPS model to explicitly re-introduce coupling (more details in section 4.1). Addition of this “coupling” potential initially resulted in numerical instability that turned out to be caused by sampling close to a singularity. A penalty potential was added to the model to remedy this (see section 3.2).

In addition to coarse-graining in terms of the bead definition, interaction potentials also need to be coarse-grained. Nonbonded interactions (electrostatic and steric) were already implemented by a single interaction site for each monosaccharide in the model by Bathe et al.,^18^ so their corresponding potentials are readily transferred onto the 1BPS model. For the bonded interactions, Boltzmann inversion is used to develop the interaction potentials between beads in the “coarse-scale” (1BPS) model based on results from the “fine-scale” (MD-Bathe) model. However, due to the presence of a coupling between DOFs in the 1BPS model, single Boltzmann inversion cannot be used to derive its potentials. Therefore, IBI is used to develop the bonded potentials for the 1BPS model.

## 3 Method

### 3.1 Iterative Boltzmann Inversion

The potentials for the angles GXG and XGX, and the dihedrals GXGX and XGXG for each GAG in the 1BPS beads are calculated using the IBI method based on the iterative scheme^21^
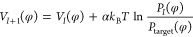
1where *V*_*I*+1_(φ) is the potential for DOF φ in iteration step *I* + 1, *P*_*I*_(φ) is an analytical fit to the probability density function (PDF) of φ in iteration step *I* and *P*_target_(φ) is an analytical fit to the PDF of φ obtained from the MD-Bathe simulation. Here φ can denote any of the angles and dihedrals listed above, and α is a convergence control parameter between 0 and 1. This iterative process starts from an initial set of potentials *V*_0_ that is taken to be *V*_0_(φ) = −α*k*_B_*T* ln *P*_target_(φ). During IBI, the nonbonded interactions are switched off.

Initially an energy minimization was done using the steepest decent method for a single chain with 16 monosaccharides. Afterward, an MD relaxation run of the 1BPS model using the set of potentials *V*_*I*_(φ) was carried out, which were used to calculate the probability densities *P*_*I*_(φ). The Langevin dynamics simulations were carried out at a temperature of 300 K by using GROMACS 2019.4 with an implicit solvent and a friction coefficient of 50 ps^–1^. These simulations were 40 ns long with a time step of 1 fs from which the trajectory of the chain was recorded every 250 fs.

The probability densities calculated from the recorded simulations need to be inter- and extrapolated to ensure continuity. Interpolation is necessary because the probabilities are calculated using histograms with finite-width bins of 1° and 7.2° for angles and dihedrals, respectively. Extrapolation, on the other hand, is necessary because angles corresponding to high potential values may not be sampled at all. For dihedrals, quadratic splines are used that are periodic and differentiable at −180° and 180°. The PDF of angles θ is fitted to the form
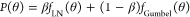
2Here β is a fitting parameter, *f*_LN_ is a log-normal distribution,
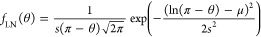
3and *f*_Gumbel_ is a Gumbel distribution,
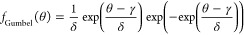
4

The value of the parameters *s*, μ, γ, and δ is determined by least-squares fitting, with the initial guess taken to be the fitted value for the target PDF. In the iterative process it was observed that in some cases, least-squares fitting could result in “poor” fits that subsequently gave rise to an instability of the IBI algorithm. This could be avoided by restricting the parameter values to a certain range. This range as well as the fitted values for the target PDF are reported in Table S1.

### 3.2 Dihedral Coupling

The method described in the previous section ensures that PDFs of angles and dihedrals in the 1BPS model match those obtained from the MD-Bathe model. However, it does not ensure that the correlation between angles and dihedrals is retained properly; therefore, we will refer to the model presented in the previous section as the “1BPS-without-coupling” model. Indeed, in section 4.1, we will demonstrate that there is a significant correlation between GXGX and XGXG dihedrals in the MD-Bathe model, which the 1BPS-without-coupling model fails to capture. To resolve this, we will introduce a systematic way to explicitly introduce coupling between the GXGX and XGXG dihedrals.

**Figure 2:**
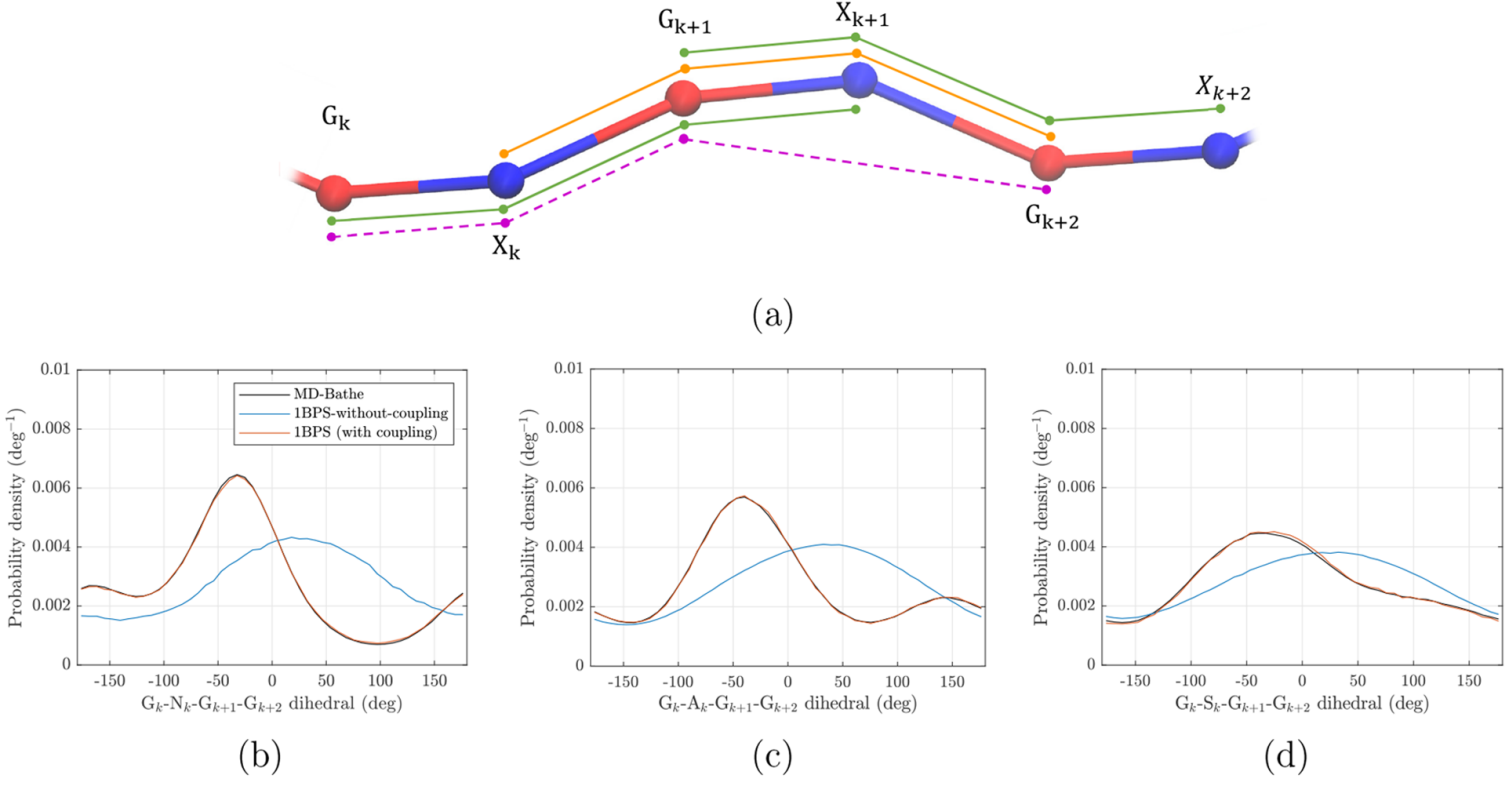
(a) Schematic representation of a part of a generic GAG chain with GXGX dihedrals shown in green and an XGXG dihedral shown in orange. The dihedral G_*k*_–X_*k*_–G_*k*+1_–G_*k*+2_ (GXGG) is shown by dashed purple lines, where *k* identifies the disaccharide unit. Two GXGX dihedrals are depicted to show all possible distances, angles, and dihedrals. (b–d) Probability density of the GXGG dihedral in the MD-Bathe model, 1BPS-without-coupling model, and 1BPS (with coupling) model for (b) HA, (c) C4S, and (d) C6S.

Figure 2a shows a schematic representation of a generic part of a chain that includes two adjacent dihedrals; the relevant beads are labeled with an index for clarity. The approach is to add extra bonded interaction(s) (bond, angle, or dihedral), which we call “coupling potential”, to the model in order to couple GXGX and XGXG dihedrals.

Consider the dihedral G_*k*_–X_*k*_–G_*k*+1_–X_*k*+1_ (which we will call GXGX) and X_*k*_–G_*k*+1_–X_*k*+1_–G_*k*+2_ (which we will call XGXG) shown in Figure 2a. We want to add a potential that couples the GXGX dihedral with the XGXG dihedral by adding a bonded interaction between a subset of beads G_*k*_, X_*k*_, G_*k*+1_, ..., G_*k*+2_. Since the G_*k*+2_ bead is not in the GXGX dihedral, it is necessary for the coupling potential to act on the G_*k*+2_ bead. This would allow the location of the G_*k*+2_ bead (therefore the value of the XGXG dihedral) to be affected by (therefore correlated with) the values of the GXGX dihedral. Similarly, the G_*k*_ bead should also be included in the coupling potential.

A bond between G_*k*_ and G_*k*+2_ is the simplest potential that could be used for coupling GXGX and XGXG. Yet, any angle or dihedral that connects G_*k*_ to G_*k*+2_ could also be used as a coupling potential between GXGX and XGXG. This will give us a list of bonds, angles, and dihedrals that can be used for coupling GXGX to XGXG. One can do a similar exercise with XGXG and the dihedral G_*k*+1_–X_*k*+1_–G_*k*+2_–X_*k*+2_ (also shown in Figure 2a). This time, the beads X_*k*_ and X_*k*+2_ are the necessary beads to be included in the coupling potential. By combining these two lists all the possible coupling potentials can be obtained.

Not all of the possible coupling potentials are equally effective. The effectiveness in coupling the two dihedrals depends on the difference between the coupled (MD-Bathe) model and uncoupled (1BPS-without-coupling) model. We compared the PDFs of distances, angles, and dihedrals from the list of possible coupling potentials between the MD-Bathe model and the 1BPS-without-coupling model. The results of this comparison can be found in Figures S6–S8. The most pronounced difference between the MD-Bathe and the 1BPS-without-coupling models is observed in the dihedral model defined by G_*k*_–X_*k*_–G_*k*+1_–G_*k*+2_ beads (or simply the GXGG dihedral) in all three GAGs. Therefore, we choose the coupling potential to correspond to the GXGG dihedral. Addition of this dihedral to the model will enforce the PDF of this dihedral to match that of the MD-Bathe model resulting in a coupling of GXGX and XGXG dihedrals. The potential for the GXGG dihedral is developed via eq 1 simultaneously with other bonded potentials discussed in section 3.1.

When the angle between three beads gets close to 180°, the dihedral force may be singular (see more in ref (22)). The instability resulting from the singularity can limit the time step of the CG model (in our case, the time step was limited to 1 fs). This scenario took place for the X_*k*_–G_*k*+1_–G_*k*+2_ (or simply the XGG) angles. In order to prevent the angle from coming close to 180°, we use the penalty potential
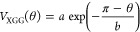
5with *a* = 100 kJ/mol and *b* = 0.026 radians. The parameter *b* was tuned such that the peaks of the probability densities of the MD-Bathe and 1BPS models occurred at about the same angle. In addition to yielding excellent agreement of the two potentials, this value of *b* allowed us to use the same time step of 20 fs as in the 1BPA model.^14^

### 3.3 Nonbonded Interactions

Similar to the model by Bathe et al.,^18^ nonbonded interactions are modeled exclusively by virtual sites. The respective potentials are the same as those used by Bathe et al.,^18^ but the location of both virtual sites has been changed to the center between adjacent beads (see Figure 1c,e). Additionally, contrary to the approach by Bathe et al.,^18^ we do allow adjacent monosaccharides in the 1BPS model to interact via nonbonded interactions; including these interactions was found to have a negligible effect on the chain conformation.

The electrostatic interaction is incorporated through a Debye–Hückel potential,
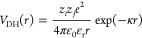
6where *z*_*i*_ is the charge of monosaccharide *i* (which is 0 for VN and −1 for all other virtual sites), *e* is the elementary charge, ε_0_ is the permittivity of vacuum, ε_r_ = 80 is the dielectric constant of water, and κ^–1^ = (ε_0_ε_r_*k*_B_*T*/(2*e*^2^*N*_A_*C*_s_))^1/2^ is the Debye length. Here *N*_A_ is Avogadro’s number, and *C*_s_ is the monovalent salt concentration.

Steric repulsion is modeled by a shifted Lennard-Jones potential,
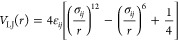
7with a cutoff distance of *r*_*ij*_ = 2^1/6^σ_*ij*_ where σ_*ij*_ = (1/2)(σ_*i*_ + σ_*j*_) and ε_*ij*_ = 0.6276 kJ/mol. Here σ_*i*_ = 0.329, 0.356, 0.356, and 0.356 nm for the VG, VN, VA, and VS sites, respectively.

## 4 Results and Discussion

### 4.1 Development of Bonded Interactions

**Figure 3:**
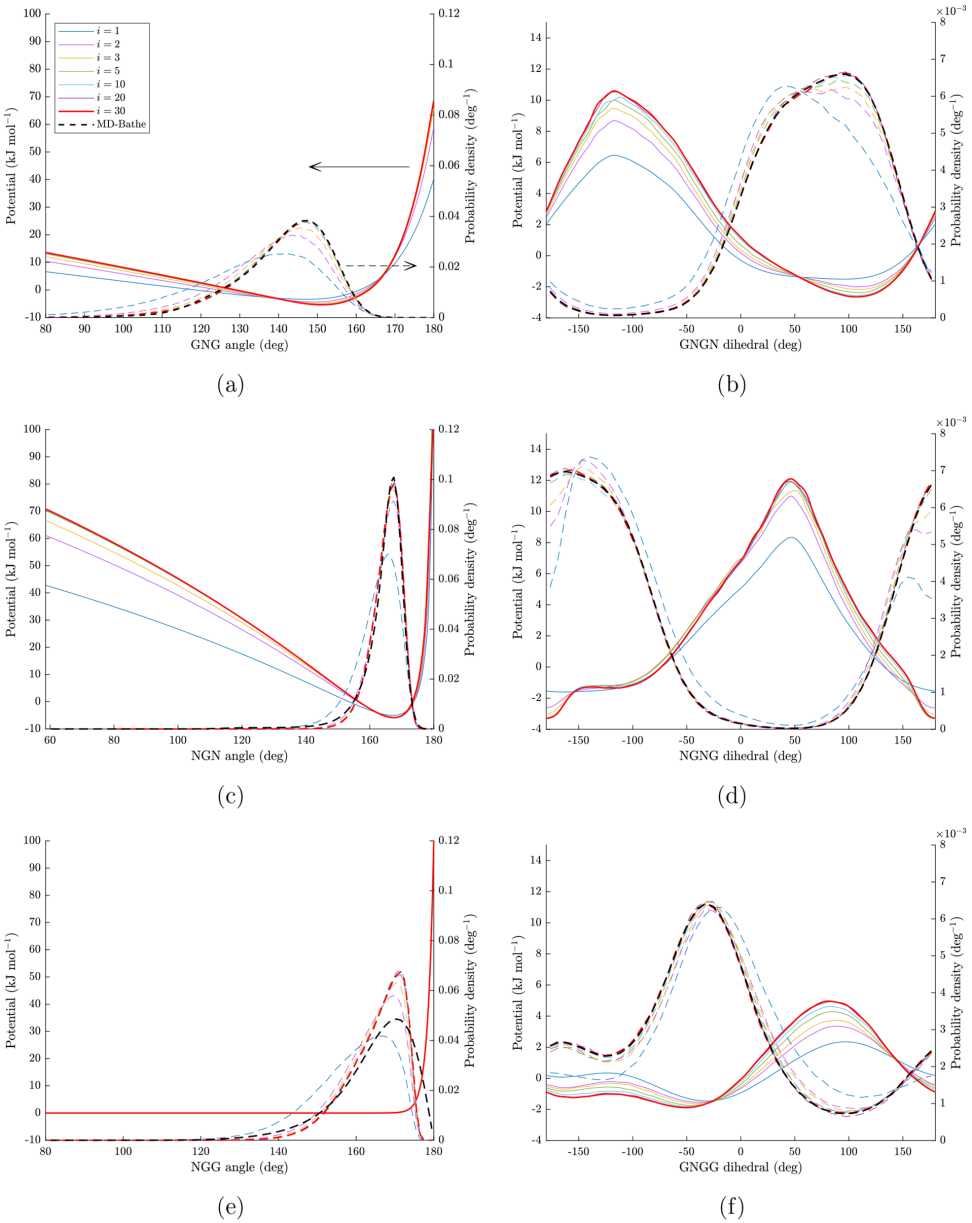
Potentials (solid lines) and probability densities (dashed lines) of angles and dihedrals for an HA chain in different steps of iterative Boltzmann inversion (IBI). The black dashed lines correspond to target probability densities calculated from the MD-Bathe model. Thick red lines correspond to PDFs and potentials in the last step of the IBI (*i* = 30). The potentials in panels a–d and f are updated iteratively using eq 1, while the potential in panel e is the penalty potential defined in eq 5 which is kept constant during IBI.

The IBI method was used to develop the bonded potentials of the 1BPS model. Figure 3 shows the potentials and probability densities of the angles and dihedrals in different iteration steps of the IBI for an HA chain. It was observed that the IBI method converged within 30 iteration steps for all GAGs (see the results for the chondroitin sulfates in Figures S4 and S5).

It should be noted that the XGG potential, eq 5, is not updated in the IBI method. The PDFs reported for these angles are calculated as an output of the IBI. The fact that the PDFs of the XGG angles for the final iteration (*i* = 30) do not match those of MD-Bathe is intentional in order that sampling close to 180° is prohibited to ensure a stable solution algorithm.

With the current method, the accuracy with which the PDFs of the angles according to MD-Bathe can be recovered is limited by how closely the analytical fit can capture the features of the PDFs in each iteration. Different analytical fit functions were tried, and the current choice, eq 2, was found to give an accurate description of the PDFs. The GROMACS-compatible potential files obtained at the end of the IBI process for angles and dihedrals are provided in the Supporting Information.

**Figure 4:**
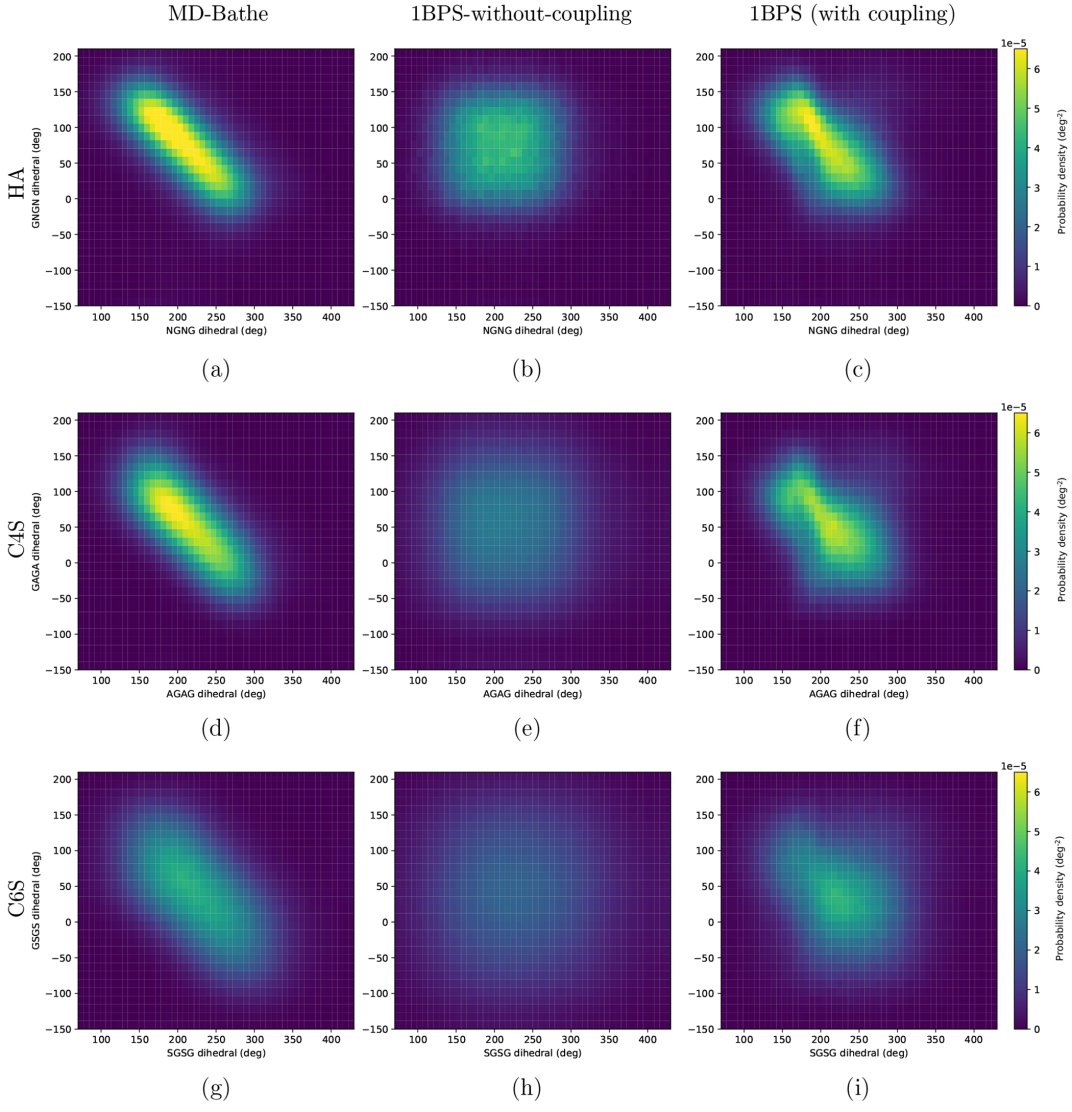
Ramachandran-type plots for probability densities of (a–c) HA, (d–f) C4S, and (g–i) C6S. Plots a, d, and g correspond to the MD-Bathe model; plots b, e, and h correspond to the 1BPS-without-coupling model, where X is N, A, or S for HA, C4S, and C6S chains, respectively. Plots c, f, and i correspond to the 1BPS (with coupling) model.

The MD-Bathe model implicitly includes a correlation between the GXGX and XGXG dihedrals as shown in the Ramachandran-type plots in Figure 4a,d,g. In the absence of any coupling potential, these two dihedrals would be independent of one another, resulting in Figures 4b,e,h. Clearly, the latter deviate strongly from Figure 4a,d,g.

Addition of the GXGG coupling potential as described in section 3.2 results in an improved correlation of the GXGX and XGXG dihedrals as shown in Figure 4c,f,i. By including this coupling potential, the absolute maximum deviation of the probability densities from the MD-Bathe model gets reduced from 3.6 × 10^–5^ deg^–2^ in the 1BPS-without-coupling model to 1.5 × 10^–5^ deg^–2^ in the 1BPS (with coupling) model, as demonstrated in Figure S9. This improvement subsequently yields a better prediction of the chain conformation. We study the chain conformation as governed by the bonded interactions in terms of the characteristic ratio,
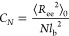
8where ⟨*R*_ee_^2^⟩_0_ is the mean squared end-to-end distance of the chain, *N* is the number of the monosaccharides of the chain, and *l*_b_ = 5.25 Å is the average length of the GX and XG bonds.

**Figure 5:**
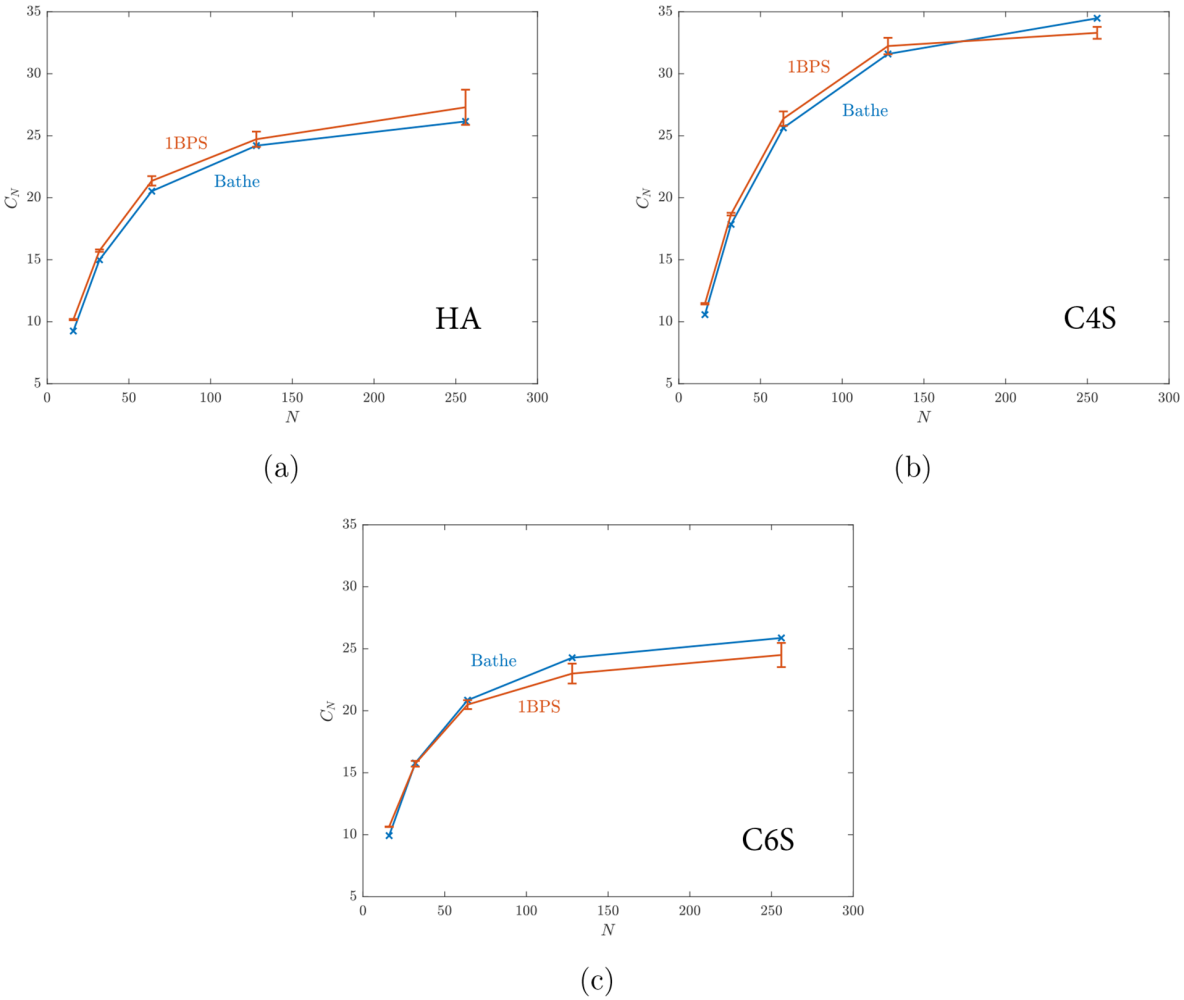
*C*_*N*_ vs number of monosaccharides, *N*, for (a) HA; (b) C4S, and (c) C6S. Every error bar shows the standard deviation of five replica simulations. We observe that the 1BPS model can capture the conformation of the chains without nonbonded interactions reported by Bathe et al.^18^ quite accurately.

Figure 5 shows that the *C*_*N*_ values predicted by the 1BPS model closely match those reported by Bathe et al.^18^ The addition of the GXGG coupling potential is not a perfect solution for the coupling of GXGX and XGXG, as Figure 4a,d,g are not completely reproduced in Figure 4c,f,i, even though the 1BPS probability densities are quite close to those from the MD-Bathe model (Figures S6–S8). One could consider trying to improve the accuracy of the model by adding other potentials in order to capture the coupling of dihedrals more accurately. For example, we attempted a GGXG potential to the current model for C6S and observed that the shape of the Ramachandran-type plot in Figure 4i approaches that of Figure 4g and that the *C*_*N*_ value for long C6S chains improves somewhat. However, addition of this potential resulted in reduced stability of the model; therefore, we decided not to include it in the 1BPS model.

The existence of correlations between DOFs in the “fine-scale” model is one of the challenges of the IBI method. These correlations are typically avoided, minimized, or ignored in coarse graining using this method^23−26^ through an appropriate choice of the mapping scheme. We have also tried other mappings than that illustrated in Figure 1 to limit the above-mentioned dihedral coupling but faced the difficulty that both the GXG and XGX angles frequently sampled 180°. Choosing the glycosidic oxygen as a mapping point, as we do in Figure 1, not only improves stability of the 1BPS model but also has the advantage that the distance between two beads has a clear physical meaning reflecting the size of a monosaccharide.

We used the penalty function in eq 5 to avoid singularities in the dihedral force. Bulacu et al.^22^ have proposed using a restricted bending potential to avoid this type of singularity. However, restricted bending potentials have a preference for a specific angle, whereas the penalty function used here is chosen such that it does not give preference for any specific angle but only serves to avoid sampling close to 180°.

### 4.2 Effect of Nonbonded Interactions

GAGs are highly charged polysaccharides, and electrostatic interactions play an important role in the conformation of these chains. In this section, we study the effect of the salt concentration (as the main factor affecting the strength of electrostatic interactions by Debye screening) on the persistence length. The specific aim is to confirm that the 1BPS model can reproduce the salt concentration dependence of the persistence length as reported by Bathe et al.^18^

The persistence length, *l*_p_, is calculated by fitting a function of the form exp(−*nl*_b_/*l*_p_) to the autocorrelation of bond vectors, *C*(*n*), separated by *n* beads.^27^ The autocorrelation, *C*(*n*), is computed from
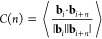
9where **b**_*i*_ is the vector connecting bead *i* to its neighbor *i* + 1.

**Figure 6:**
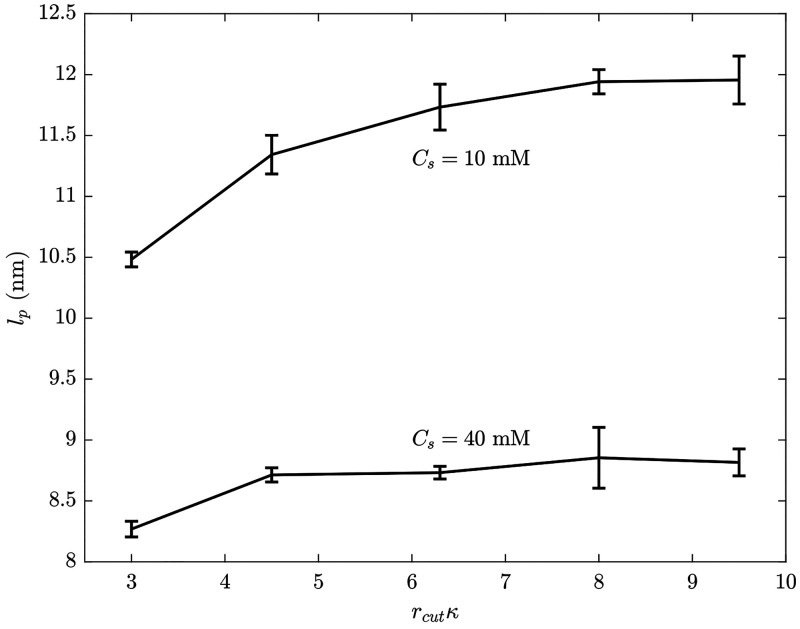
Persistence length vs cutoff distance normalized by Debye length κ^–1^ for an HA chain with 128 monosaccharides at two salt concentrations. Every error bar shows the standard deviation of five replica simulations.

In general, a cutoff distance is adopted to reduce the computational cost of a model, with an acceptable trade-off for accuracy. Bathe et al.^18^ used a cutoff distance for electrostatic interactions of 3 times the Debye length κ^–1^. However, when using this cutoff distance in the 1BPS model we found that the predicted persistence length of a chain with 128 monosaccharides is underestimated at low salt concentrations, as shown in Figure 6. We recommend to use a cutoff distance of 8 times the Debye length in our model, since the persistence length has converged at this cutoff distance. This cutoff distance is recommended for modeling individual chains in the range of salt concentrations covered in the current manuscript. For other simulations (e.g., interactions between multiple chains, other salt concentrations), a study similar to the one shown in Figure 6 may be needed to arrive at an appropriate or optimized value of the cutoff distance.

**Figure 7:**
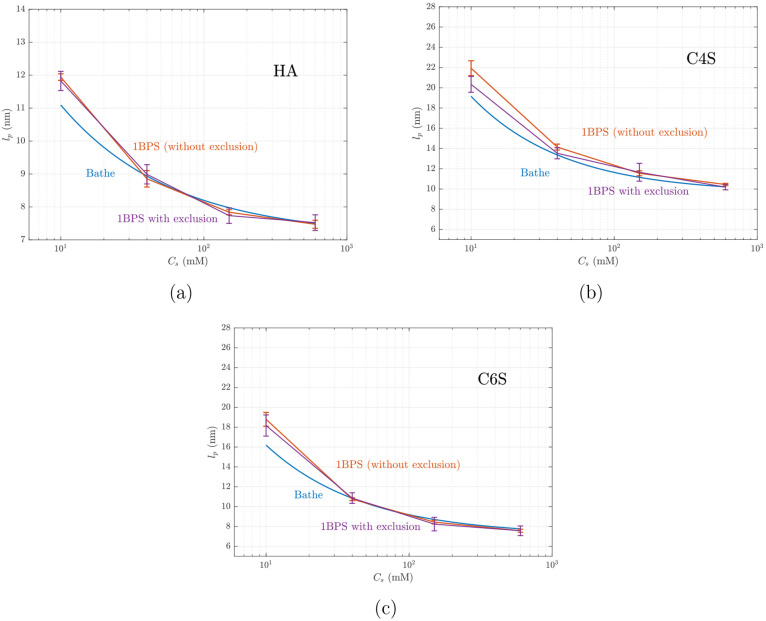
Persistence length vs salt concentration predicted by the 1BPS model (with and without excluding interactions between adjacent monosaccharides) vs the reported result by Bathe et al.^18^ for (a) HA; (b) C4S, and (c) C6S. All GAGs have 128 monosaccharides, and the 1BPS simulations are carried out with a cutoff distance of 8κ^–1^, whereas Bathe et al.^18^ have used 3κ^–1^. Every error bar shows the standard deviation of five replica simulations.

Figure 7 shows the 1BPS predictions of the persistence length compared to the values reported by Bathe et al.^18^ We observe good agreement at high salt concentrations, where electrostatic interactions are relatively weak. In this figure, we also demonstrate that the exclusion of adjacent monosaccharides from nonbonded interactions, as adopted in ref (18), indeed has a small effect on the persistence length. In combination with Figure 5, these results confirm that coarse-graining of the bonded interactions was carried out properly. At a salt concentration of *C*_s_ = 10 mM the two models predict different values, which can be traced back to differences in the cutoff distance, as discussed above (see Figure 6). The 1BPS model tends to slightly overestimate the persistence length of the C4S chains; this slight difference might be related to including nonbonded interactions between adjacent monosaccharides in the 1BPS model.

The coarse-graining proposed here allows us to use a time step of 20 fs compared to a maximum time step of 1 fs in the MD-Bathe model. This means that we have developed a model that has 3 times fewer DOFs and is 20 times faster. However, this was done at the cost of introducing two extra (GXGG and XGG) potentials. In addition, the defined angle potentials are not captured analytically but need to be tabulated. By comparing two sets of simulations that produced the same results using MD-Bathe and the 1BPS model, we quantified the speedup obtained by the 1BPS model to be ∼24.

**Figure 8:**
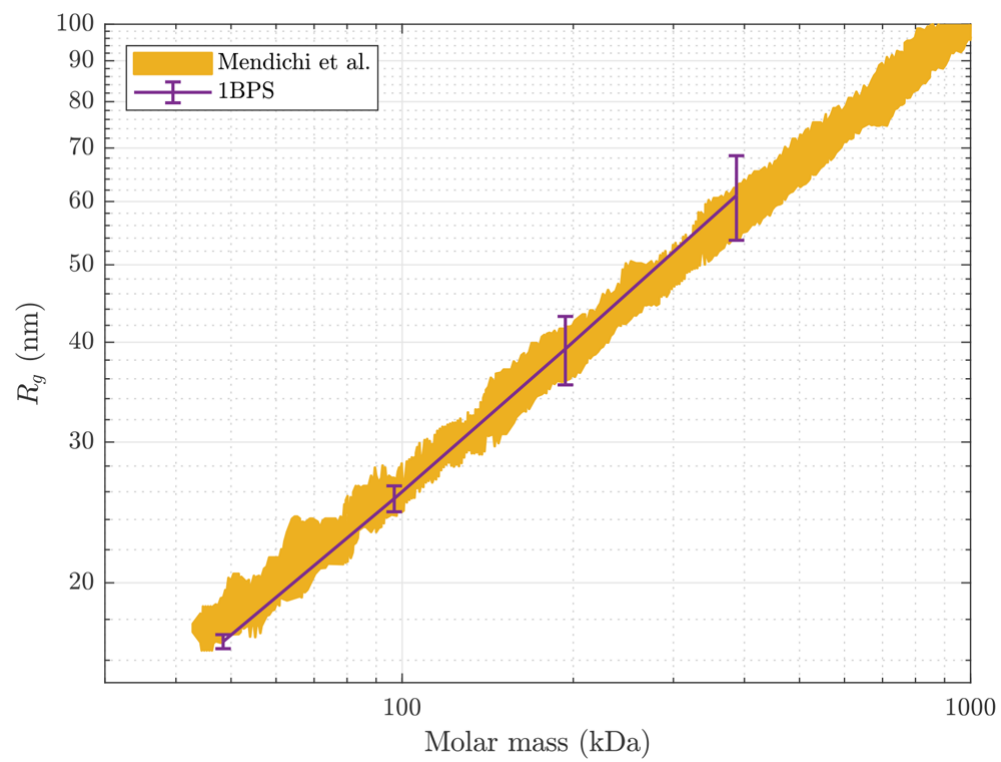
Radius of gyration vs molar mass of HA chains at a 150 mM NaCl salt concentration. Every error bar shows the standard deviation of five replica simulations. The simulated chains have a molar mass of 48.4, 96.8, 193.6, and 387.2 kDa which correspond to 256, 512, 1024, and 2048 monosaccharides, respectively. The yellow region corresponds to the experimental data by Mendichi et al.^28^

Coarse-graining also allowed us to model longer GAGs. Figure 8 shows the 1BPS predictions of the radius of gyration *R*_g_ as a function of the molar mass of HA, revealing a close match with the experimental observations by Mendichi et al.^28^ In these simulations, we were able to predict the conformation of HA chains with as many as 2048 monosaccharides (corresponding to a molecular mass *M* of 387.2 kDa). The 1BPS model predicts a power law *R*_g_ ∝ *M*^ν^ with ν = 0.63 ± 0.03 (95% confidence interval). The exponent of this power law is overestimated by only ∼5% compared to ν = 0.6 reported experimentally.^28^

Obviously, next to the aforementioned computational advantages, the 1BPS model has its limitations in terms of resolution. This is partly due to the reduction of the number of degrees of freedom, which leads to a GX bond length of 5.6 Å defining the spatial resolution of the 1BPS representation of GAGs. The resolution is also inherently limited by the use of an implicit solvent, as this excludes the explicit incorporation of hydrogen bonding, hydrophobicity, multivalency of ions, and charge distribution. Like in the original model by Bathe et al.,^18^ the effects of the first two of these are implicit in the 1BPS model through its potentials. The actual distribution of counterions near the charge sites in the chains is invisible due the mentioned resolution. Hence, the arguments given by Bathe et al.^18^ in favor of using the Debye–Hückel potential carry over to the 1BPS model.

### 4.3 Conclusion

In this article, we developed a 1BPS model for modeling GAGs. Coarse-graining was carried out starting from an existing model^18^ that was first converted from a Monte Carlo into an MD model. We used iterative Boltzmann inversion to coarse-grain the bonded interactions while introducing an explicit coupling between the dihedral angles. The adopted methodology for coupling dihedrals can be used as a generic approach to explicitly introduce correlations between degrees of freedom in other systems, as well.

The focus has been on the development of a 1BPS force field for HA and chondroitin sulfate chains. The methodology has been described in sufficient detail that it can be adopted for developing 1BPS models for other GAGs, such as dermatan sulfates, keratan sulfates, and heparan sulfates, which also play important roles in biology.^1^ As the proposed methodology does not depend on the type of polymer, it may even be used for developing one bead per monomer models of other intrinsically disordered polymers.

It bears emphasis that our approach is targeted at coarse-graining the distribution functions of the relevant degrees of freedom. Intra- and intermolecular forces are derived subsequently from the corresponding coarse-grained potentials. Force matching is an alternative approach in which the parameters in preselected CG potentials are fitted so as to minimize the deviation of the corresponding forces from the finer-scale (atomistic) forces. Common force-matching methods are aimed primarily at increasing the computational efficiency by considering relatively simple coarse-grained potentials, yet iterative methods are under development to improve the accuracy in molecules with more complex angular and dihedral interactions.^29^ It remains to be seen if such a method can also efficiently deal with the coupling between degrees of freedom, as they occur in GAGs.

## Supporting Information Available

Additional details of potentials and results for the MD-Bathe and the 1BPS model: Example of calculation of the MD-Bathe glycosidic dihedral potential for ψ_1,3_ dihedral for HA (Figure S1); mean squared end-to-end distance for HA chain as a function of maximum value of dihedral potential in the MD-Bathe model (Figure S2); dihedral potentials used in the MD-Bathe model (Figure S3); ranges and fitted values for the parameters of analytical fits for angle potentials for each GAG (Table S1); potentials and PDFs of angles and dihedrals for C4S chain (Figure S4) and C6S chain (Figure S5) in different steps of IBI; Comparison of PDFs of distances, angles, and dihedrals that have the potential for coupling GNGN and NGNG dihedrals (Figure S6), GAGA and AGAG dihedrals (Figure S7), and GSGS and SGSG dihedrals (Figure S8) between MD-Bathe, 1BPS (with coupling) and 1BPS-without-coupling for C6S chain; deviation of 1BPS-without-coupling and 1BPS (with coupling) Ramachandran-type plots from the MD-Bathe model (Figure S9); Description of the content of GROMACS-compatible potential files (PDF)
GROMACS-compatible potential files for the angles and dihedrals for the 1BPS model (ZIP)
