# Supplementary material for: A One-Bead-Per-Saccharide (1BPS) Model for Glycosaminoglycans
Source: J Chem Theory Comput. 2023 Jul 17;19(16):5491–502. doi: 10.1021/acs.jctc.3c00238 (PMC10448712; doi:10.1021/acs.jctc.3c00238)
Supplement: Supplementary file 1 — ct3c00238_si_001.pdf [file ct3c00238_si_001.pdf]

Supporting Information: A one-bead-per-saccharide (1BPS) model for glycosaminoglycans

Saber Shakibi, Patrick R. Onck, and Erik Van der Giessen*

Micromechanics of Materials, Zernike Institute for Advanced Materials, University of Groningen, 9747 AG Groningen, The Netherlands

E-mail: E.van.der.giessen@rug.nl

1 MD-Bathe model

The bonded potentials in the MD-Bathe model to replace the rigid bonds in the original Monte Carlo description[S1] are

$$V_{\text{bond}}(r_{ij}) = \frac{1}{2}k_b(r_{ij} - r_{ij}^0)^2 \tag{1a}$$

$$V_{\text{angle}}(\theta_{ijk}) = \frac{1}{2}k_a(\theta_{ijk} - \theta_{ijk}^0)^2 \tag{1b}$$

$$V_{\text{dihedral}}(\xi_{ijkl}) = k_d(1 + \cos(\xi_{ijkl} - \xi_{ijkl}^0)) \tag{1c}$$

where k_b (= 15000 kJ mol^{-1} nm^{-2}), k_a (= 5000 kJ mol^{-1} rad^{-2}) and k_d (= 5000 kJ mol^{-1}) are the stiffness of the bond, angle and dihedral potential; r_{ij}, θ_{ijk} and ξ_{ijkl} are distance, angle and dihedrals in nm, radians and radians, respectively; r_{ij}^0, θ_{ijk}^0 and ξ_{ijkl}^0 are taken

from the equilibrium distance, angle and dihedrals from Table 2 of the supplementary material of Ref.[S1]

Figure S1 illustrates the procedure for obtaining the glycosidic dihedral potentials for the MD-Bathe model based on the $V(\psi_{1,3})$ data. These potentials are obtained with cubic spline fitting to potential data points $V(\psi_{1,3})$ obtained from the PMF data of Bathe et al.[S1] The spline fitting is carried out in such a way that differentiability is ensured at the boundaries.

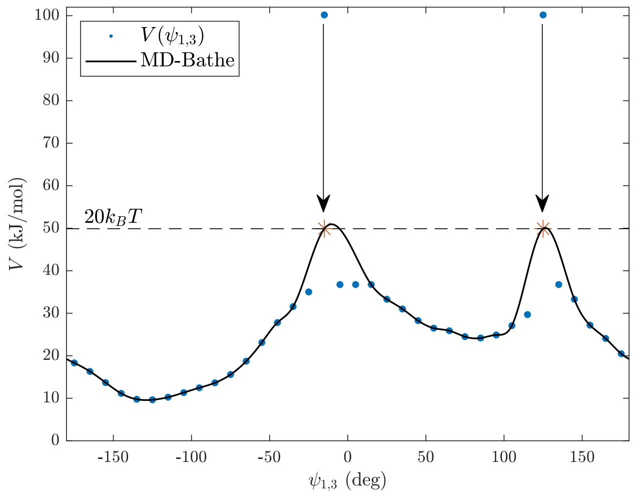

Figure S1: MD-Bathe glycosidic dihedral potential for $\psi_{1,3}$ dihedral (black line) obtained from cubic spline fitting to $V(\psi_{1,3})$ data (blue data points) for HA. Data points above the 20 k_BT (dashed line) are moved to 20 k_BT (star points) as shown by the vertical arrows. Some data points are ignored to improve smoothness of the fit.

In the fitting procedure, we use a maximum value of the potential that is low enough to allow for sampling in between peaks. The effect of the maximum value of the glycosidic dihedral potential on the mean squared end-to-end distance of HA chains in absence of non-bonded interactions is shown in Figure S2. It is seen that a maximum potential of 20 k_BT is sufficient for a correct prediction of chain conformation in the MD-Bathe model, whereas higher values tend to overestimate the characteristic ratio as certain orientations are effectively excluded from sampling.

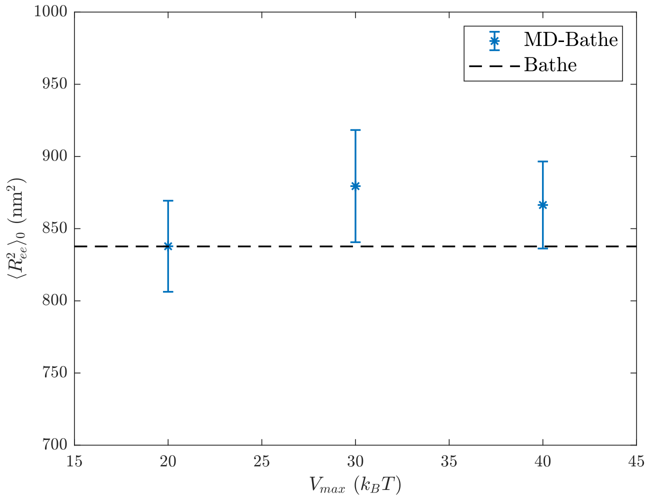

Figure S2: Mean squared end-to-end distance, $\langle R_{ee}^2 \rangle_0$, for HA chain with 128 monosaccharides as a function of the maximum value of the dihedral potential in the MD-Bathe model. The dashed line is the value reported by Bathe et al.[S1] These results are obtained with all non-bonded interactions switched off.

Glycosidic dihedral potentials used in MD-Bathe are shown in Fig. S3. These potentials are obtained using the procedure described in Section 2.1. Virtual sites for the steric and electrostatic interactions are specified through the 3out method in GROMACS, such that the location of the virtual sites matches those described in Table 2 of the supplementary material of Ref.[S1]

2 xvg files for potentials

The potential files obtained from the IBI algorithm are provided in a zip file as GROMACS-compatible .xvg files. These files correspond to the angles GXG, XGX and XGG as well as dihedrals GXGX, XGXG and GXGG for each chain. Since the XGG potential is the same for all GAGs, one generic file is provided for this potential. The first column of this file contains the values of angles φ in degrees, the second column contains the value of potentials $V(\varphi)$ in kJ/mol and the third column contains the negative derivative of the

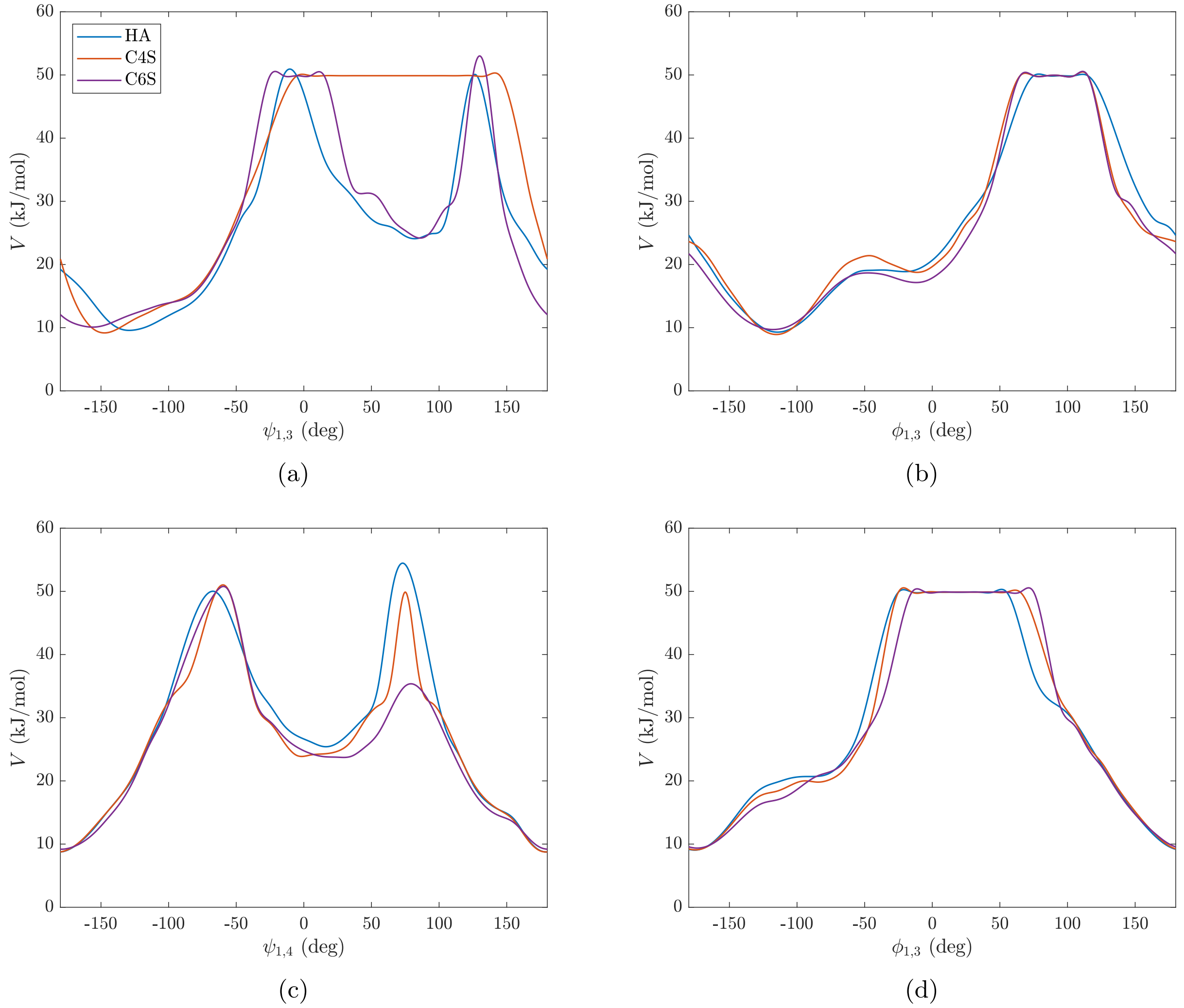

Figure S3: Dihedral potentials for $\psi_{1,3}$ (a), $\phi_{1,3}$ (b), $\psi_{1,4}$ (c) and $\phi_{1,4}$ dihedrals (d) for HA, C4S and C6S used in the MD-Bathe model.

potentials ($-\mathrm{d}V(\varphi)/\mathrm{d}\varphi$) in kJ mol^{-1} deg^{-1}.

3 Supplementary figures and tables

References

(S1) Bathe, M.; Rutledge, G. C.; Grodzinsky, A. J.; Tidor, B. A Coarse-Grained Molecular Model for Glycosaminoglycans: Application to Chondroitin, Chondroitin Sulfate,

Table S1: Values of the parameters for the analytical fit for the angles. The 'Target' values are obtained from least squares fitting of the data obtained from MD-Bathe model. The 'Min' and 'Max' values specify the two boundaries for the fitting (values $\pm\infty$ indicate that no limitations are imposed). For XGX angles, a lognormal fitting sufficiently describes the distribution, $\beta = 1$, and the Gumbel parameters γ, δ are not applicable (NA). The parameters β and s are dimensionless. The value of μ reported here is compatible with angles expressed in radians; for working in degrees, the value of μ should be increased by $\ln(180/\pi)$.

Parameter		GNG	NGN	GAG	AGA	GSG	SGS
	Min	0.0	1.0	0.0	1.0	0.0	1.0
β	Target	0.04453	1.0	0.5070	1.0	0.5373	1.0
	Max	1.0	1.0	1.0	1.0	1.0	1.0
	Min	2.0	$-\infty$	2.0	$-\infty$	2.0	$-\infty$
s	Target	3.421	2.643	3.264	2.642	3.479	2.694
	Max	4.0	∞	4.0	∞	4.0	∞
	Min	−3.948	$-\infty$	−3.948	$-\infty$	−3.948	$-\infty$
μ	Target	−3.886	−3.744	−3.868	−3.737	−3.595	−3.714
	Max	−3.548	∞	−3.548	∞	−3.548	∞
	Min	0.0	NA	0.0	NA	0.0	NA
γ (rad)	Target	2.552	NA	2.592	NA	2.726	NA
	Max	∞	NA	∞	NA	∞	NA
	Min	0	NA	0.0	NA	0.0	NA
δ (rad)	Target	0.1739	NA	0.1929	NA	0.08488	NA
	Max	∞	NA	∞	NA	∞	NA

and Hyaluronic Acid. *Biophysical Journal* **2005**, *88*, 3870–3887.

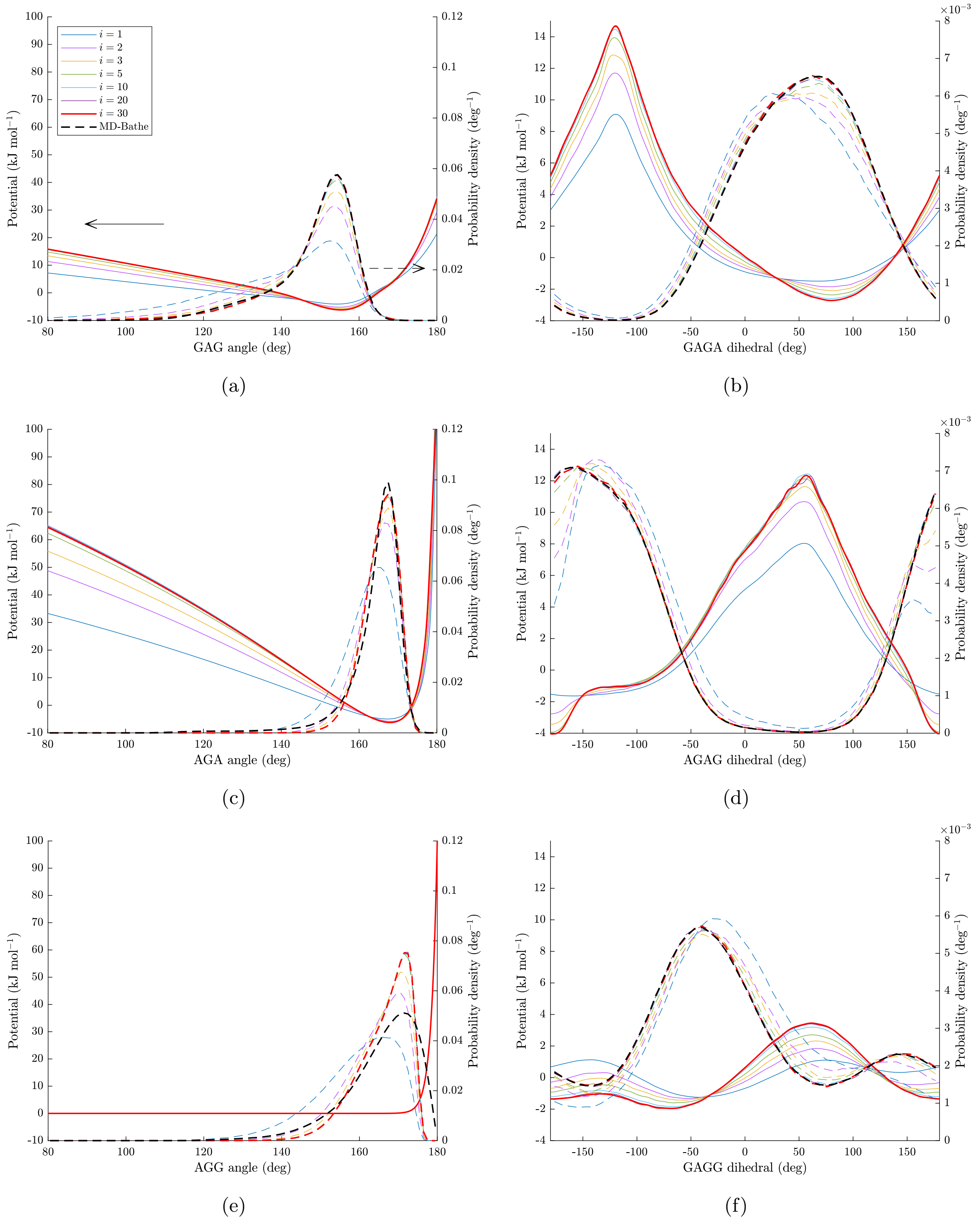

Figure S4: Potentials (solid lines) and probability densities (dashed lines) of angles and dihedrals for C4S chain in different steps of iterative Boltzmann inversion (IBI). The black dashed lines correspond to target probability densities calculated from the MD-Bathe model. Thick red lines correspond to PDFs and potentials in the last step of IBI (i = 30). The potentials in (a–d) and (f) are updated iteratively using eq 1 while the potential in (e) is the penalty potential defined in eq 5 which is kept constant during IBI.

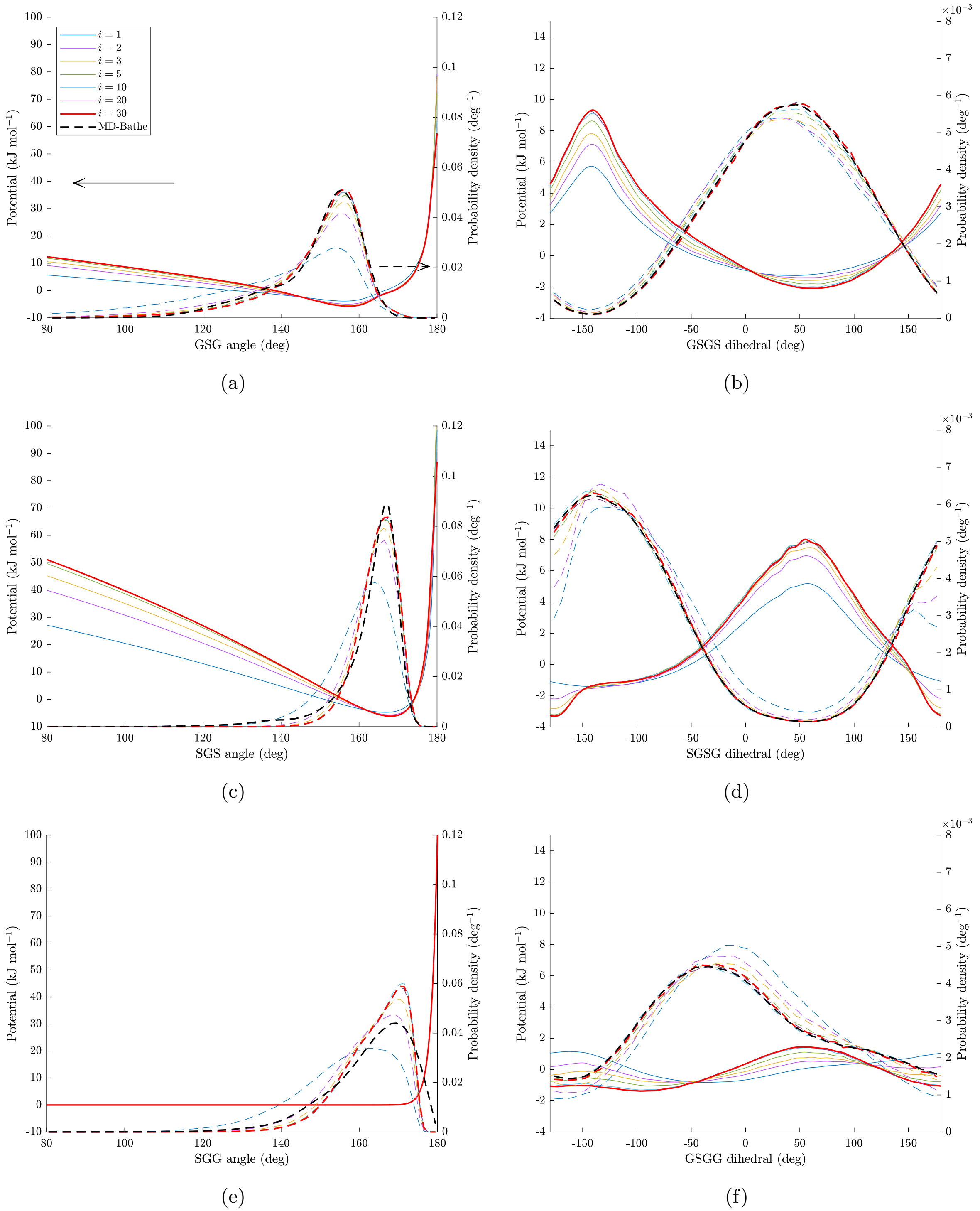

Figure S5: Potentials (solid lines) and probability densities (dashed lines) of angles and dihedrals for C6S chain in different steps of iterative Boltzmann inversion (IBI). The black dashed lines correspond to target probability densities calculated from the MD-Bathe model. Thick red lines correspond to PDFs and potentials in the last step of IBI ($i = 30$). The potentials in (a–d) and (f) are updated iteratively using eq 1 while the potential in (e) is the penalty potential defined in eq 5 which is kept constant during IBI.

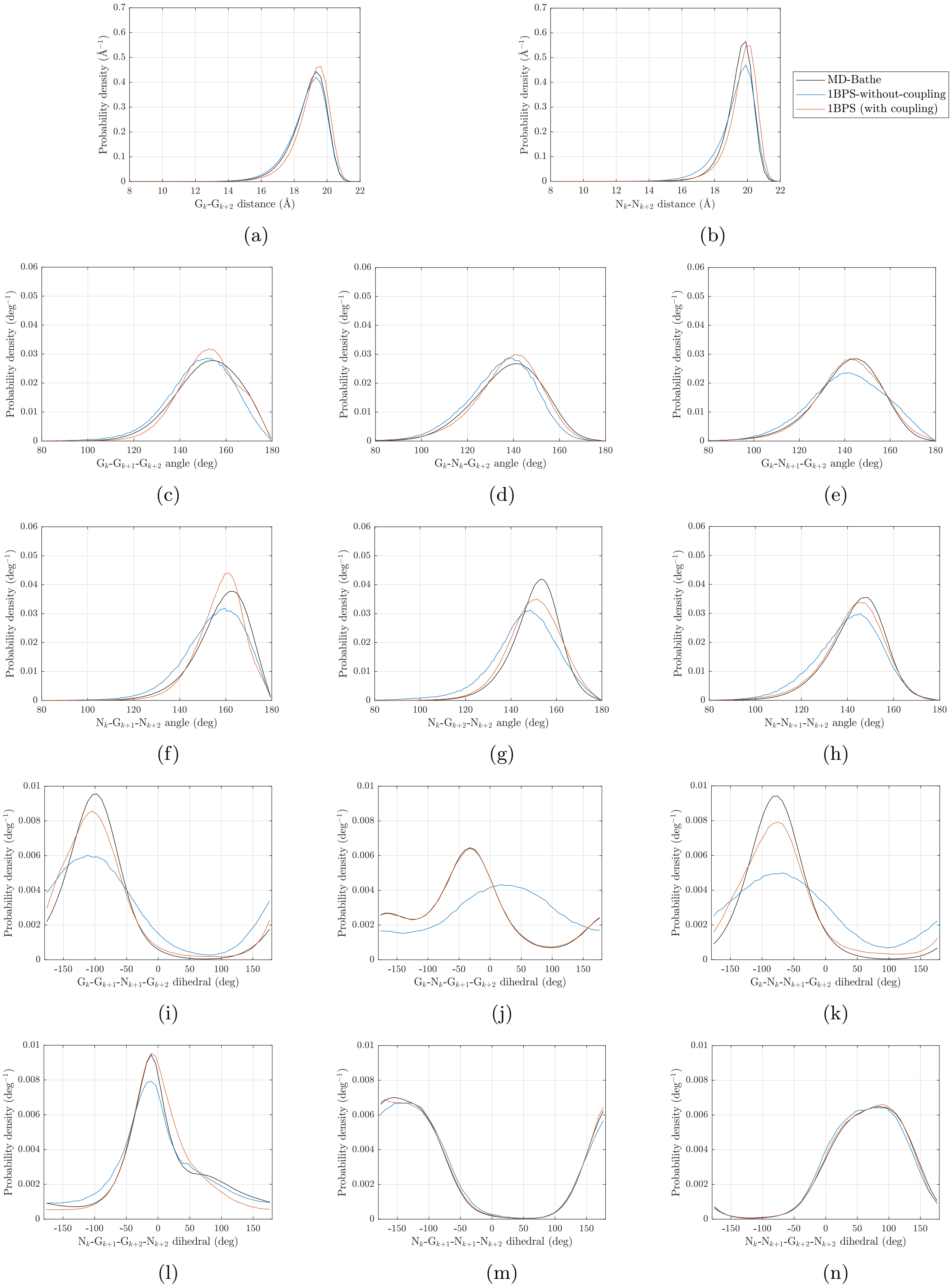

Figure S6: Comparison of PDFs of different distances, angles and dihedrals between MD-Bathe, 1BPS (with coupling) and 1BPS-without-coupling for an HA chain with 16 monosaccharides. For the definition of these DOFs, see Figure 2a.

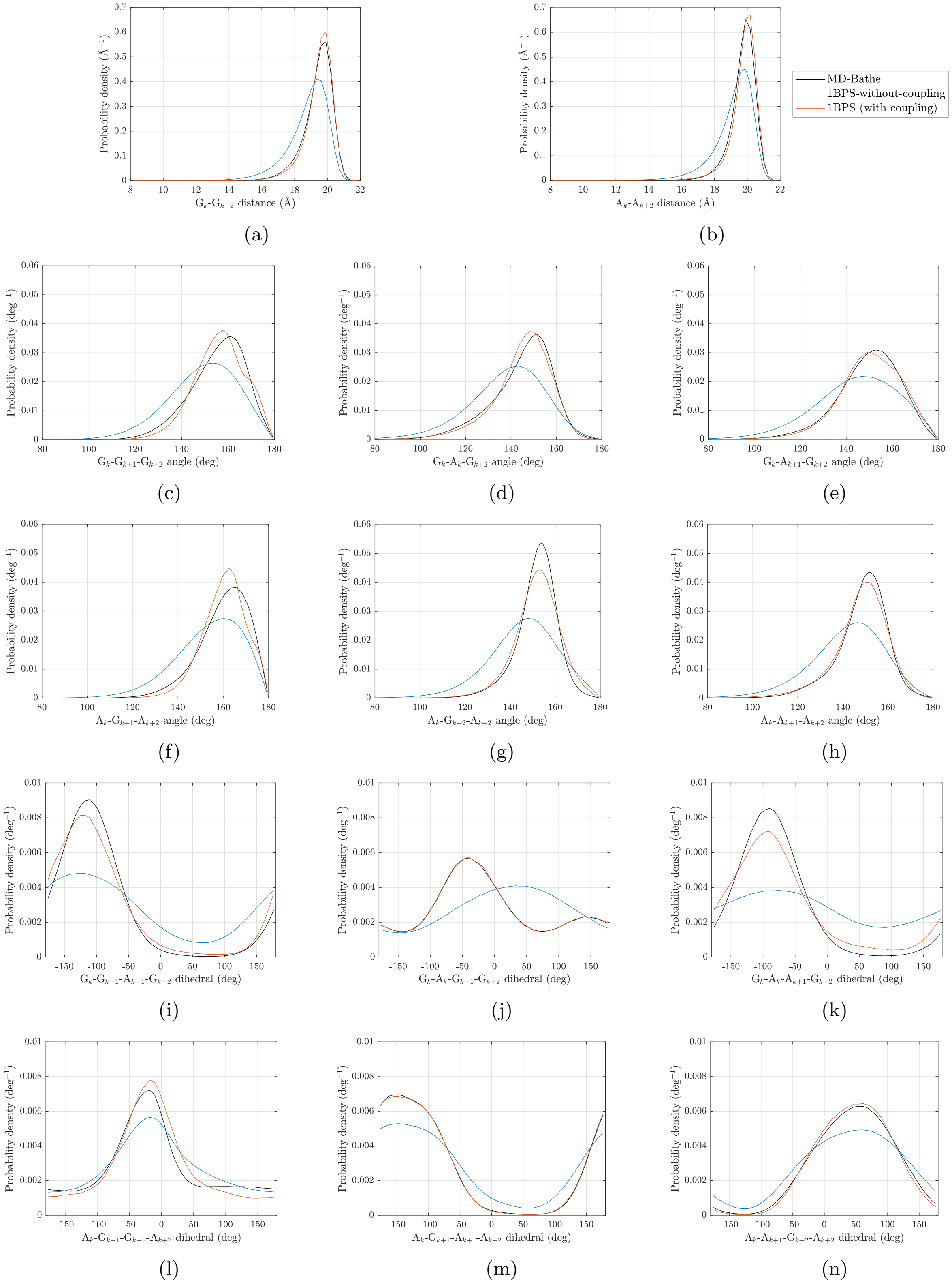

Figure S7: Comparison of PDFs of different distances, angle and dihedrals between MD-Bathe, 1BPS (with coupling) and 1BPS-without-coupling for a C4S chain with 16 monosaccharides. For the definition of these DOFs, see Figure 2a.

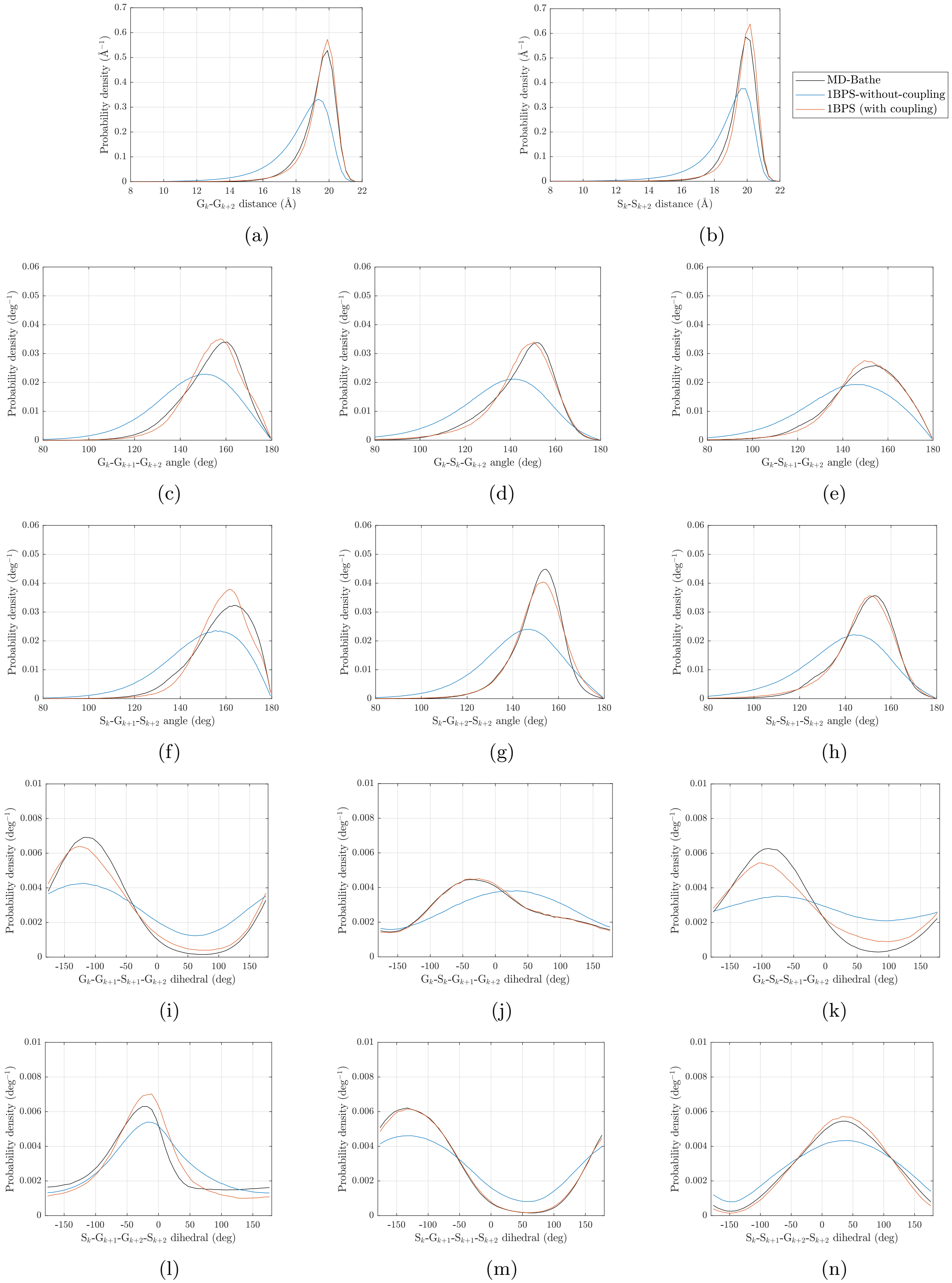

Figure S8: Comparison of PDFs of different distances, angle and dihedrals between MD-Bathe, 1BPS (with coupling) and 1BPS-without-coupling for a C6S chain with 16 monosaccharides. For the definition of these DOFs, see Figure 2a.

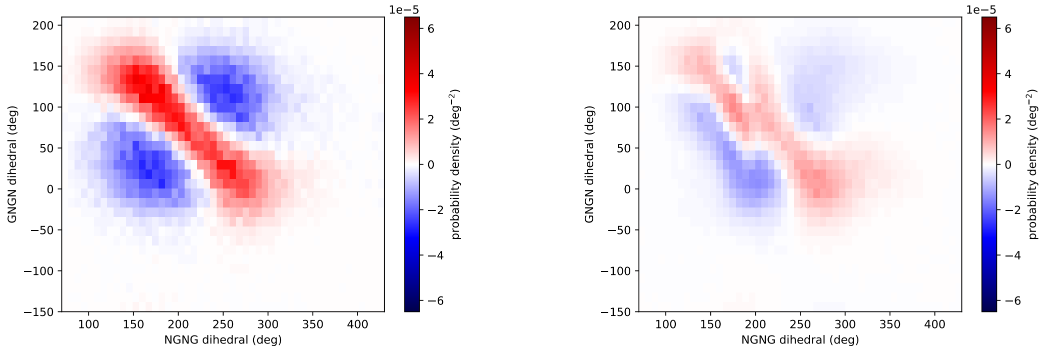

(a) (b)

Figure S9: The difference in probability densities of HA (shown in Fig. 4) compared to the MD-Bathe model (a) for the 1BPS-without-coupling model and (b) the 1BPS (with coupling) model. The absolute maximum deviation was decreased from (a) 3.6×10^{-5} deg^{-2} to (b) 1.5×10^{-5} deg^{-2}.
